# Hypophosphatasia: The Unusual Presentation

**DOI:** 10.4274/jcrpe.galenos.2020.2020.0094

**Published:** 2020-11-25

**Authors:** Consolato M. Sergi

**Affiliations:** 1 Stollery Children’s Hospital and Department of Laboratory Medicine and Pathology, University of Alberta, Edmonton, Canada

**Keywords:** Hypophosphatasia, corneal opacity, band keratopathy, neonatal presentation

## Dear Editor,

Esmel-Vilomara et al (1) describe the unique case of an infant with an extraordinary presentation of hypophosphatasia with corneal opacity diagnosed shortly after birth during clinical investigation. The authors found a previously unreported mutation, found in the *ALPL* gene encoding TNAP. It was a heterozygous variant, c.1292T>A found in exon 11 of the *ALPL* gene, producing an amino acid change p.(Val431Asp). The authors suggest that the underlying etiology may explain the mild phenotype of this case of hypophosphatasia using bioinformatics tools. The newborn presented with a transient band keratopathy, which can occur in the setting of hypercalcemia (2,3). Hypercalcemia can trigger band keratopathy, and this disorder can be transient. Band keratopathy is a corneal disease that originated from the appearance of calcium deposits on the central cornea. This aspect is a notable example of metastatic calcification, which occurs in hypercalcemia. Transient band keratopathy has been described in patients with systemic hypercalcemia. The etiology includes pituitary disturbances, renal failure, and sarcoidosis. Nevertheless, neonatologists should consider keeping in mind that another presentation may be more common in hypophosphatasia, i.e., the Vitamin B6 dependent seizure. Vitamin B6-dependent seizures include a group of treatable diseases (*ALDH7A1* deficiency, *PNPO* deficiency, *PLP* binding protein deficiency, hyperprolinemia type II, hypophosphatasia, and glycosylphosphatidylinositol anchor synthesis defects) responding to pyridoxine or pyridoxal-5I-phosphate (4). Baumgartner-Sigl et al (4) presented a 7-month-old girl, who presented as a neonate with pyridoxine-responsive seizures but without bone abnormalities. She had initial normal cognitive milestones but later failed to thrive. Nearly undetectable serum ALP activity, elevated plasma PLP and urinary phosphoethanolamine and inorganic pyrophosphate levels, hypercalcemia, hypercalciuria, and nephrocalcinosis were consistent with infantile hypophosphatasia. Sequence analysis of the *TNAP* gene revealed missense mutations in exon 7 (c.677T>C, p.M226T) and exon 10 (c.1112C>T, p.T371I). Overall, the clinical presentation of hypophosphatasia remains highly variable, ranging from perinatal death to adult osteopenia and dental problems. There are six subtypes of hypophosphatasia, including lethal perinatal, benign perinatal, infantile, childhood, adult, and odontoid-hypophosphatasia with the lethal perinatal hypophosphatasia being the most severe (5). Babies born with this condition show rapidly worsening alterations of calcium/phosphate metabolism (hypercalcemia), apneic spells, seizures, and progressive encephalopathy that may occasionally mimic a hypoxic-ischemic encephalopathy. Despite considered rare, hypophosphatasia affects all races around the world, with a highly variable incidence and a particularly high prevalence in the United States and Canada. Thus, Esmel-Vilomara et al’s (1) patient addresses the important aspect of the clinical screening for metabolic disorders in routine clinical examination of babies.
